# Longitudinal Analysis of Fecal Microbiome and Pathologic Processes in a Rotenone Induced Mice Model of Parkinson’s Disease

**DOI:** 10.3389/fnagi.2017.00441

**Published:** 2018-01-08

**Authors:** Xiaodong Yang, Yiwei Qian, Shaoqing Xu, Yanyan Song, Qin Xiao

**Affiliations:** ^1^Department of Neurology, Ruijin Hospital, Shanghai Jiao Tong University School of Medicine, Shanghai, China; ^2^Department of Biostatistics, Institute of Medical Sciences, Shanghai Jiao Tong University School of Medicine, Shanghai, China

**Keywords:** fecal microbiota, 16S rRNA gene sequencing, rotenone, inflammation, Parkinson’s disease

## Abstract

Recent studies reported an association between gut microbiota composition and Parkinson’s disease (PD). However, we know little about the relationship between microbiome dysbiosis and the pathogenesis of PD. The objective of this study was to describe the evolution of fecal microbiota using an oral rotenone model of PD from a longitudinal study over a period of 4 weeks. Gastrointestinal function was assessed by measuring fecal pellet output, motor functions was assessed by open-field and pole tests every week. α-synuclein pathology, inflammation and tyrosine hydroxylase (TH) neuron loss from the middle brain were also analyzed. Fecal samples were collected every week followed by 16S rRNA sequencing and bioinformatics analysis. We reported that chronically oral administered rotenone caused gastrointestinal dysfunction and microbiome dysbiosis prior to motor dysfunction and central nervous system (CNS) pathology. 16S rRNA sequencing of fecal microbiome showed rotenone-treated mice exhibited fecal microbiota dysbiosis characterized by an overall decrease in bacterial diversity and a significant change of microbiota composition, notably members of the phyla Firmicutes and Bacteroidetes, with an increase in Firmicutes/Bacteroidetes ratio after 3 weeks of rotenone treatment. Moreover, rotenone-induced gastrointestinal and motor dysfunctions were observed to be robustly correlated with changes in the composition of fecal microbiota. Our results demonstrated that gut microbiome perturbation might contribute to rotenone toxicity in the initiation of PD and brought a new insight in the pathogenesis of PD. Novel therapeutic options aimed at modifying the gut microbiota composition might postpone the onset and following cascade of neurodegeneration.

## Introduction

Parkinson’s disease (PD) is a neurodegenerative disorder estimated to affect more than 1% of the over-65 population. The neuropathological hallmarks of PD are loss of dopaminergic neurons in the substantia nigra and the presence of cytoplasmic inclusions called Lewy bodies in the remaining surviving dopaminergic neurons (Kalia and Lang, 2015). It has become increasingly evident that PD could also affect several neuronal structures outside the substantia nigra, among which is the enteric nervous system (ENS), according with this pathological features, there is high frequency of various gastrointestinal (GI) symptoms in patients with PD. Constipation serves as the most prominent GI dysfunction of PD is estimated as a premotor symptom before the classical motor symptoms (Fasano et al., 2015). Functional and structural changes of gastrointestinal tissues have been found in PD patients, such as accumulation of α-synuclein in the ENS (Shannon et al., 2012), impaired colonic mucosa barrier function (Devos et al., 2013) and bacterial invasion (Fasano et al., 2013). Lewy pathology manifests in enteric neurons of the gut long before it is present in dopaminergic neurons of the midbrain and PD symptoms are evident (Braak et al., 2003). The pathology accompanies with these symptoms are consistent with the Braak staging system: α-synuclein could spread from the gastrointestinal tract to the brain.

Gut is an important rout of entry for putative pathogenic environmental factors to initiate the pathological of PD. These have led to many speculations about a possible etiological role of alterations in gut-brain interactions in PD (Mulak and Bonaz, 2015). The gut-brain axis interactions are significantly modulated by the gut microbiota via immune, neural, and endocrine pathways (Wang and Kasper, 2014). Changes in the gut microbiota composition may cause alterations in the gut barrier function and intestinal permeability, affecting not only GI epithelial cells and immune system, but also play an important role in modulating behaviors and brain functions, including stress responsiveness, emotional behavior, pain modulation, ingestive behavior, and brain biochemistry (Dinan and Cryan, 2017; Rieder et al., 2017; Xu et al., 2017). The effects of environmental factors on gut microbiome community structures are not well-established yet.

Links between *Helicobacter pylori* infection, small intestinal bacterial overgrowth (SIBO), and PD particularly related to motor function and fluctuations have been demonstrated previously (Tan et al., 2014; Çamci and Oguz, 2016). It is only until the next-generation sequencing technologies for us to explore the alterations in the gut microbiota composition in PD. There is recent evidence from case-control studies both found a significant differences between PD patients and controls. However, most of the differentially abundant taxa as well as associations of microbiota with clinical variables differed between studies due to confounder from human samples (Scheperjans, 2016). What is more, the causal relationship between the microbiota changes and the pathogenesis of PD remains unclear as studies in humans have largely been limited to cross sectional analyses. In addition, study of the gut microbiota in individuals with PD occurs primarily after disease is already manifest, limiting the ability to investigate changes in the microbiota that occur early in the disease process. There is a paucity of knowledge regarding the evolution of the gut microbiota and its relationship with the pathology in the progression of PD. Notwithstanding, such information is crucial to better understand the relationship between the microbiota and the pathogenesis of PD and thus improve its prevention, diagnosis and treatment.

In order to examine the association between fecal microbiota and PD, we characterized the succession change of fecal microbiota with an ideal model of PD by oral rather than systemic administration of rotenone which is more likely to recapitulate the exposure to pesticide that may occur in normal life (Klingelhoefer and Reichmann, 2015) from a longitudinal study. We found fecal microbiota dysbiosis preceded the major motor dysfunction and central nervous system (CNS) pathology in rotenone induced PD mice model, which indicated fecal microbiota perturbation might contribute to rotenone toxicity in the initiation of PD.

## Materials and methods

### Animal experiments

**Figure 1:**
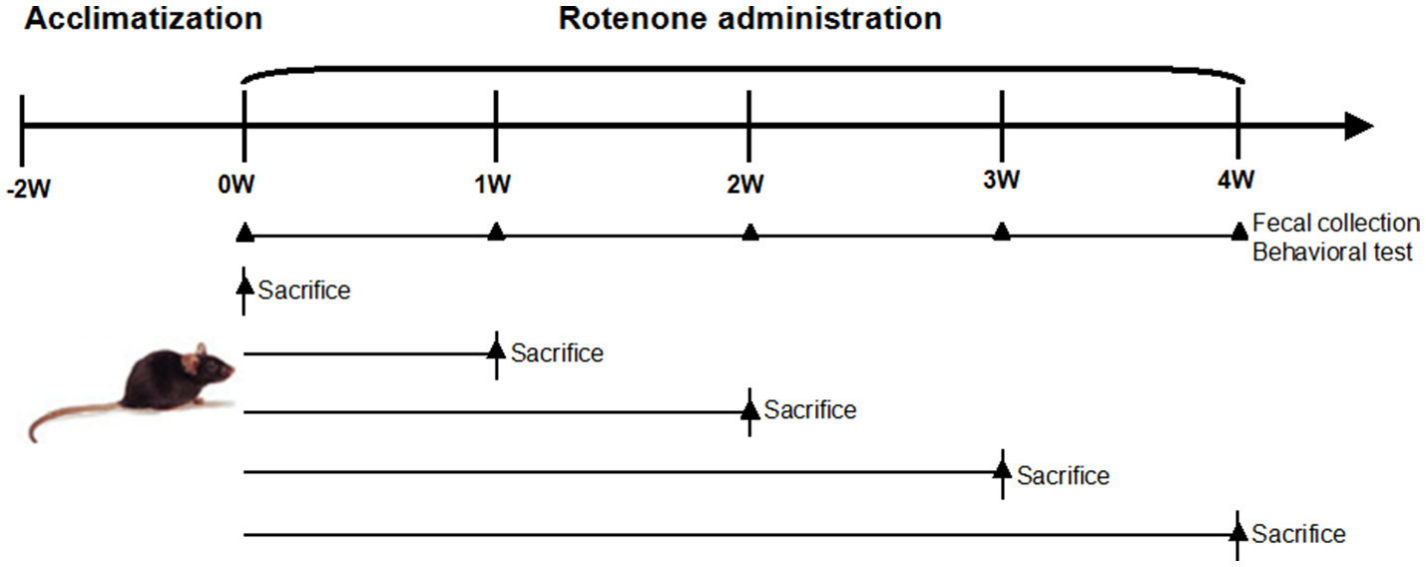
Schematic of oral rotenone administration regimen and measurable outcomes. Male C57BL/6 mice were acclimatized 2 weeks before the experiments. Mice received once daily oral rotenone administration. One group (*n* = 5) was for *in vivo* tests (fecal pellet collection, open field test, and pole test) weekly during the period. The other five groups (*n* = 6–8 per group) were sacrificed at the indicated time point, colon, and brain tissues were collected for biochemical examinations.

Male C57BL/6 mice aged between 8 and 9 weeks were purchased from Shanghai SiLaike (SLAC) Laboratory Animal Company (Shanghai, China). The mice were kept under environmentally controlled conditions (ambient temperature: 20 ± 2°C; humidity: 50–65%) on a 12 h light/dark cycle and provided *ad libitum* access to food and water. The schematic of oral rotenone administration regimen and measurable outcomes is depicted in Figure 1. Briefly, after the acclimation phase, mice were randomly divided into 6 groups, each group has 5–8 mice. One group was used for continuous observation of gut microbiota and behavioral features for 4 weeks. The other groups were sacrificed at the indicated time point for biochemical and histological tests (Figure 1). Rotenone (Sigma, St Louis, USA) was administered once daily by gavage 30 mg/kg for 4 weeks, a treatment regimen known to produce dopaminergic neuronal degeneration and α-synuclein aggregation in the CNS and GI dysfunction (Inden et al., 2007; Tasselli et al., 2013). All experiments were performed in accordance with the guidelines established by the National Institutes of Health for the care and use of laboratory animals. The protocol for carrying out animal experiments was approved by Animal Care Committee of Shanghai Jiao Tong University School of Medicine.

### Fecal sample collection and DNA extraction

Fecal samples were collected before (0 week) and 1, 2, 3, 4 weeks after rotenone treatment. Samples were collected directly from the animal upon defecation and immediately frozen at −80°C, prior to DNA extraction. Microbial genomic DNA was extracted from 200 mg of each fecal sample using a TIANamp Stool DNA Kit (Spin Column, Cat. no. DP328) according to the manufacturer’s recommendation. Successful DNA isolation was confirmed by agarose gel electrophoresis.

### Amplification, sequencing of 16S rRNA gene, and data analysis

The V4, V5 hypervariable regions of 16S rRNA gene were amplified by PCR using the barcoded derivatives of primers 520F (AYTGGGYDTAAAGNG) and 926R (CCGTCAATTYYTTTRAGTTT). The 16S rRNA gene amplicons were then sequenced using the paired-end method on the Illumina Miseq platform according to manufacturer’s instructions. 16S rRNA amplification and sequencing service were provided by Personal Biotechnology Co., Ltd. (Shanghai, China). The bacterial α-diversity were studied by biodiversity indexes including ACE, Chao, Shannon’s and Simpson’s indexes computed from the number of OTUs. β-diversity were calculated using weighted UniFrac distance and displayed using principal coordinate analysis (PCoA). Analysis of Similarities (ANOSIM) was used to evaluate group differences. Partial least-square discriminant analysis (PLS-DA), implemented in Metabo Analyst, was performed to discriminate the microbial community profiles among groups (Xia and Wishart, 2011). Permutational multivariate analysis of variance (PERMANOVA) was employed to determine the significance of microbial community partitioning due to experimental treatments based on the observed Unifrac distance matrix relative to 10,000 randomly rearranged distance matrices. Phylogenetic Investigation of Communities by Reconstruction of Unobserved States (PICRUSt) was used to predict the Kyoto Encyclopedia of Genes and Genomes (KEGG) pathways (Langille et al., 2013).

### Measurement of colon motility by one-hour stool collection

One-hour stool frequency was measured in the normal initial status, and after rotenone treatment weekly for five times. Assays were performed between 9:00 and 11:00. Each animal was removed from its home cage and placed in a clean, clear plastic cage without food or water for 1 h. Stools were collected immediately after expulsion and placed in sealed 1.5 ml tubes. The total stools were weighed to obtain a wet weight, then dried overnight at 65°C and weighed again to obtain a dry weight. The stool water content was calculated from the difference between the wet and dry stool weights. Results were normalized to body weight (Li et al., 2006; Greene et al., 2009).

### Behavioral test

The open-field test was used to evaluate spontaneous locomotor activity of animals (Walsh and Cummins, 1976). The pole test was adapted from the protocol previously described (Ogawa et al., 1985), total time required to climb down the pole was measured. Each animal performed 3 successive trials. The average of the three trials was calculated for statistical analyses.

### Quantitative real-time PCR

Quantitative analysis of inflammatory cytokine (TNF-α, iNOS, IL-6) and Toll-like receptor 2 (TLR2) were performed using primers specific for the genes by quantitative real-time PCR (qRT-PCR). Total RNA was isolated from the tissue samples and reverse transcribed into cDNA using a TaKaRa 1st-strand kit. Reactions were performed according to the manufacturer’s protocol. cDNA synthesized was subjected to real-time PCR assays with specific primers and SYBR@Premix Ex TaqTM qPCR SuperMix (Takara, Dalian, China). The assays were performed on the ABI7500 real-time PCR machine with the two-step amplification protocol: denaturation 30 s at 95°C, followed by 40 cycles of 95°C for 5 s and 60°C for 60 s. All mRNA quantification data were calculated using the Ct method and normalized to the GAPDH, presented as fold change over control.

### Elisa assays

Serum levels of inflammatory cytokine (TNF-α, iNOS, IL-6) were evaluated using commercially available Elisa kit (Shanghai Elisa Biotech Inc., China). Elisa was performed as recommended by the manufacturer. The optical density of each well was determined using a microplate reader at 405 nm, and the amounts of cytokines were calculated from the standard curve.

### Western blotting

After the mice were sacrificed at the indicated time point, the distal colon and the middle brain substantia nigra were rapidly removed and lysed in RIPA lysis buffer (50 mM Tris–HCl, pH 8.0; 1% NP-40; 0.5% sodium deoxycholate; 150 mM NaCl; 0.1% SDS) containing protease and phosphatase inhibitor cocktails and 1 mM phenyl-methylsulphonyl fluoride (PMSF). Equal amounts of protein (30 μg) were loaded per lane and separated by 10% SDS-PAGE. Proteins in the gels were transferred to immobilon polyvinylidene difluoride (PVDF) membrane. The membrane was blocked with 3% BSA and incubated with primary antibody overnight at 4°C (anti-α-synuclein, Abcam, 1:1,000; anti-TH, Santa Cruz, 1:500; anti-phospho-α-synuclein (phospho S129), Abcam 1:100; anti-MyD88, Cell Signaling, 1:1,000; anti-NF-κB, Santa Cruz, 1:1,000; anti-β-actin, Sigma, 1:5,000) followed by secondary antibodies conjugated to horseradish peroxidase (Santa Cruz Biotechnology, CA, USA) and visualized using chemiluminescent detection methods (Amersham Company).

### Immunofluorescence analysis

Immunofluorescence of tyrosine hydroxylase (TH) was performed on 14 mm paraformaldehyde fixed frozen brain sections. Slides were blocked for 60 min and incubated at 4°C overnight with anti-TH primary antibody. After washing three times with PBS, the sections were then incubated with the fluorescent secondary antibody. Images were captured using a Zeiss confocal microscope (Zeiss, LSM780, Germany).

### Statistical analysis

Analyses were conducted with SPSS 19.0 software. Data were expressed as the mean ± SEM. Molecular biology results were subjected to one-way analysis of variance (ANOVA), followed by *post-hoc* Dunnett multiple comparison tests. Microbiome and behavioral data were using repeated-measures analysis of variance (RMANOVA), followed by LSD test. Spearman correlations (2-tailed) were applied to associate differential abundant taxa with gastrointestinal and motor functions. *p* < 0.05 was accepted as statistically significant.

## Results

### Effect of rotenone on gastrointestinal and motor functions

**Figure 2:**
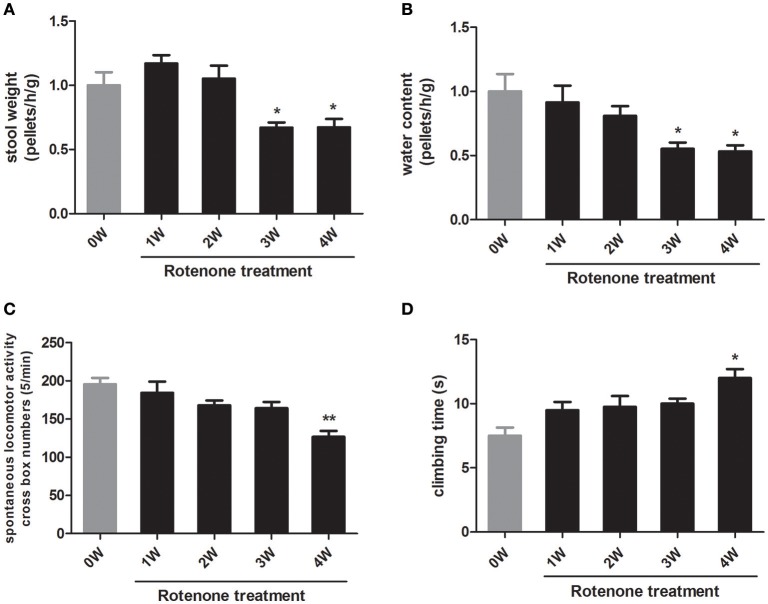
Longitudinal analysis of gastrointestinal and motor functions in rotenone-treated mice. **(A)** Fecal pellets in 1 h were collected and then recovered to initial status (0 week, baseline) **(B)** Water content of fecal pellets was calculated and then recovered to 0 week. **(C)** Spontaneous horizontal locomotor activity was determined by open-field test. **(D)** Motor coordination was determined by pole test. Data are expressed as means ± SEM of 5 mice, ^*^p < 0.05, ^**^p < 0.01 vs. 0W.

With respect to the normal initial status (0 week), there was no obvious difference in colon motility as assessed by one-hour stool weight in rotenone-treated mice at 1 week and 2 weeks. There was a decrease in colon motility at 3 and 4 weeks compared with 0 week (Figure 2A). Stool water content, another indicator of colon function, was also decreased after 3 weeks of rotenone treatment (Figure 2B), indicating mice started to show gastrointestinal dysfunction at 3 weeks.

To investigate the motor function in rotenone treated mice, we performed open-field and pole tests. Compared with the normal initial status, rotenone-treated animals exhibited locomotor deficits in open field and pole tests only at 4 weeks of rotenone treatment (Figures 2C,D). These results indicated that rotenone causes gastrointestinal dysfunction prior to the occurrence of major motor defects.

### Effect of rotenone on inflammation and α-synuclein pathology

**Figure 3:**
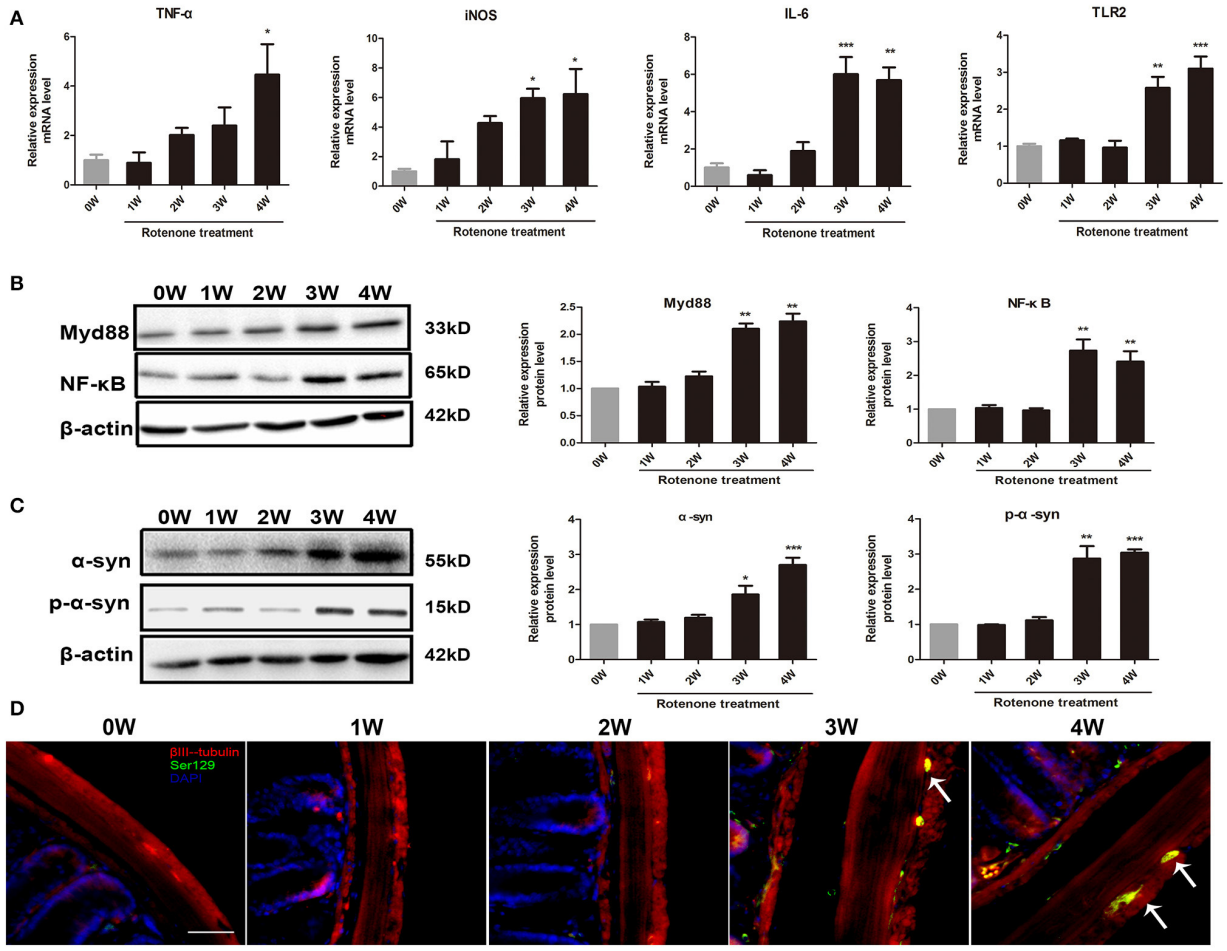
Progressive increase in inflammation and α-synuclein pathology in the colon of rotenone-treated mice. **(A)** Inflammatory cytokines (TNF-α, iNOS, IL-6) and TLR2 mRNA level in colon as determined by real-time RT-PCR. **(B)** Representative blots of Myd88 and NF-κB in colon from rotenone-treated mice. **(C)** Representative blots of α-syn and Ser129 α-syn (p-α-syn) in colon from rotenone-treated mice. **(D)** Representative photomicrographs of colon sections immunostained for anti-βIII-tubulin, anti phospho-α-synuclein (Ser 129), and DAPI staining in rotenone-treated mice (the presence of phospho-α-synuclein staining was shown by the arrows). Scale bar = 50 μm. Data are expressed as means ± SEM of at least 3 mice, ^*^p < 0.05, ^**^p < 0.01, ^***^p < 0.001 vs. 0W.

**Figure 4:**
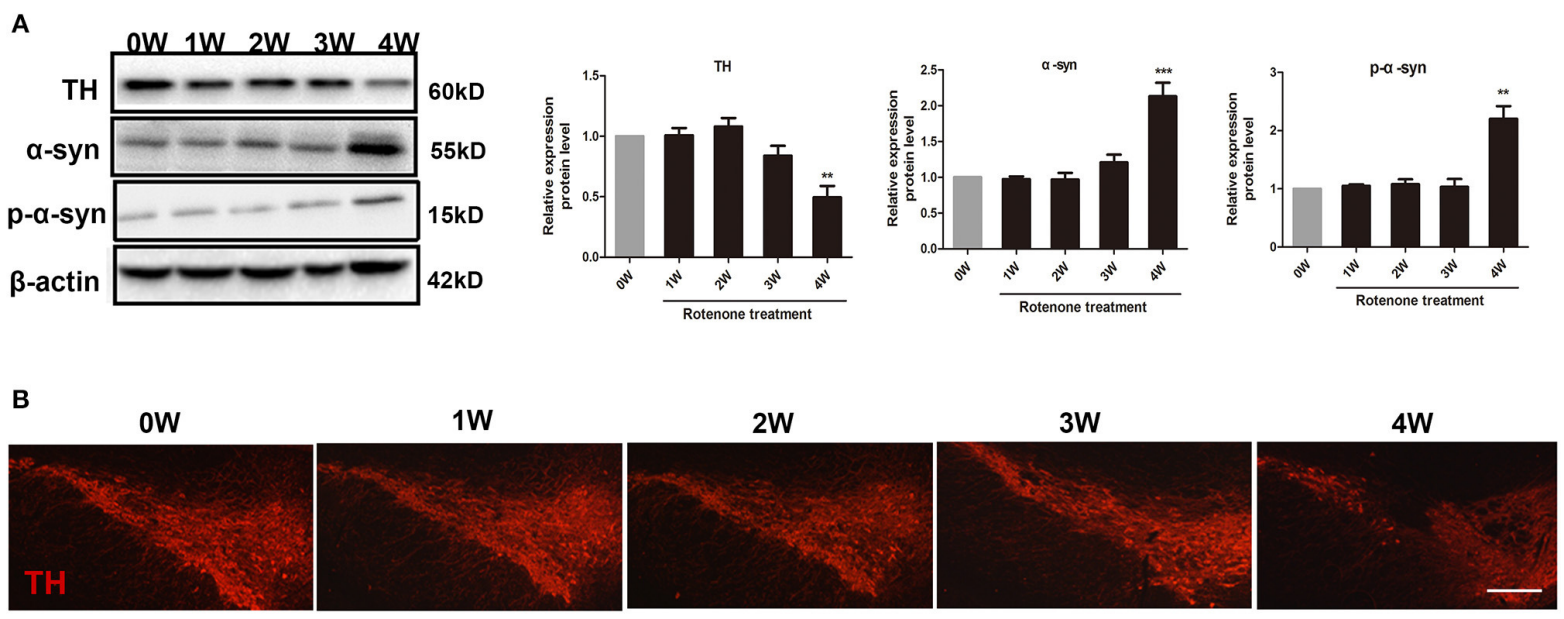
Progressive increase in α-synuclein pathology and decrease in tyrosine hydroxylase (TH) neurons in rotenone-treated mice. **(A)** Representative blots of α-syn, Ser129 α-syn (p-α-syn), and TH in the midbrain from rotenone-treated mice. **(B)** Representative photomicrographs of mesencephalic sections immunostained for TH in rotenone-treated mice. Scale bar = 500 μm. Data are expressed as means ± SEM of at least 3 mice, ^**^p < 0.01, ^***^p < 0.001 vs. 0W.

Compared with the normal initial status (0 week), we observed an inflammatory associated reaction in the colon, with increased expression of inflammatory cytokine (TNF-α, iNOS, IL-6) and toll-like receptor 2 (TLR2) in rotenone-treated mice and reaching significance at 3 and 4 weeks of rotenone treatment (Figure 3A). Since most TLR signaling pathways stimulated by mechanical ventilation culminate in the activation of the nuclear factor-κB (NF-κB). We then evaluated the expression of downstream TLR2 signaling. We found that the protein levels of myeloid differentiation factor 88 (Myd88) and NF-κB were significantly higher in the colon of rotenone-treated mice after 3 weeks of rotenone treatment (Figure 3B). There was neither significant difference in inflammatory cytokine levels (TNF-α, iNOS, IL-6) in peripheral blood by Elisa not that in the midbrain by qRT-PCR after rotenone treatment (data not shown). Increased expression of α-synuclein and Ser129 α-synuclein were detected in the colon of rotenone-treated mice from 3 weeks onward (Figure 3C). Associated α-synuclein phosphorylation was also confirmed by the presence Ser-129 phosphorylated α-synuclein in the colon section by immunofluorescence staining (Figure 3D). While increased expression of α-synuclein, Ser129 α-synuclein and reduced expression of TH were only detected at 4 weeks of rotenone treatment in the midbrain (the same time that motor deficits were detected) (Figure 4A). These were also associated with a significant decrease in TH-immunoreactive neurons in the substantia nigra as shown in representative photomicrographs (Figure 4B).

### Effect of rotenone on the overall structure of fecal microbiome

To determine if rotenone treatment could cause change in microbial diversity, fecal samples were collected weekly and the fecal bacterial population was surveyed by 16S rRNA gene sequencing. A total of 1,400,030 sequences were obtained across all samples and an average coverage of 56,001 sequences per sample. Sequences were clustered into 3,757 operational taxonomic units (OTUs) (excluding singletons) based on 97% sequence similarity.

**Figure 5:**
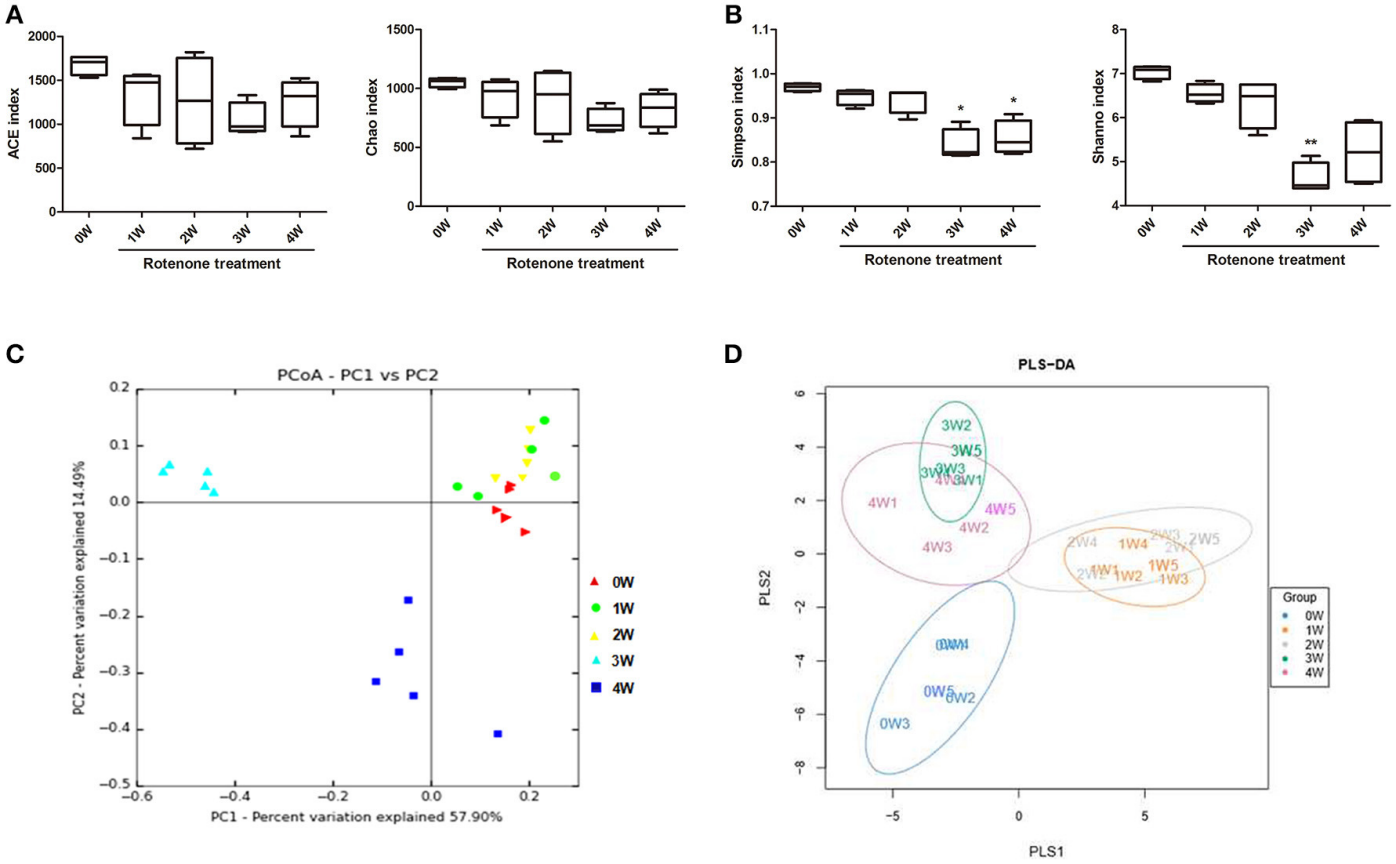
Variation in fecal bacterial α-diversity and β-diversity over time. **(A)** ACE and Chao indexes. **(B)** Simpson’s and Shannon’s diversity indexes **(C)** PCoA analysis based on weighted Unifrac distance. **(D)** Score plots of PLS-DA among the microbial community data in rotenone-treated mice. ^*^p < 0.05, ^**^p < 0.01 vs. 0W.

The microbial diversity and species richness were assessed by α-diversity metrics on the OTU data including ACE, Chao1, Simpson’s and Shannon’s diversity indexes. There were no difference among time points with regard to ACE and Chao indexes of the gut microbiota (Figure 5A). There was a decrease in Simpson’s and Shannon’s diversity indexes at 3 weeks and a decrease in Simpson’s diversity index at 4 weeks of rotenone exposure compared with the baseline (Figure 5B), indicating a disruption of the fecal microbiota.

The β-diversity analysis indicates the extent of similarity between microbial communities by measuring the degree to which membership or structure is shared between communities. To evaluate the β-diversity changes in gut microbiota across groups, principal coordinate analysis (PCoA) was applied to the whole dataset of bacterial counts based on the weighted Unifrac distance matrixes. The microbiota can separate into three groups based on sampling time. The microbiota at 3 weeks clustered in the upper-left part of the plot, 4 out of 5 mice at 4 weeks clustered in the lower left part of the plot, while the fecal microbiota of before rotenone treatment and at weeks 1 and 2 clustered in the upper-right part of the ordination and could not be divided into separated clusters (Figure 5C). Using the anosim test, only 3 weeks (*p* < 0.01) and 4 weeks (*p* < 0.05) yielded a significant difference compared with the normal initial status (0 week). Partial least-square discriminant analysis (PLS-DA), implemented in Metabo Analyst, was performed to discriminate the microbial community profiles among groups. Score plots showed that the groups were well-separated (Figure 5D).

### Effect of rotenone on the fecal microbiota composition

**Figure 6:**
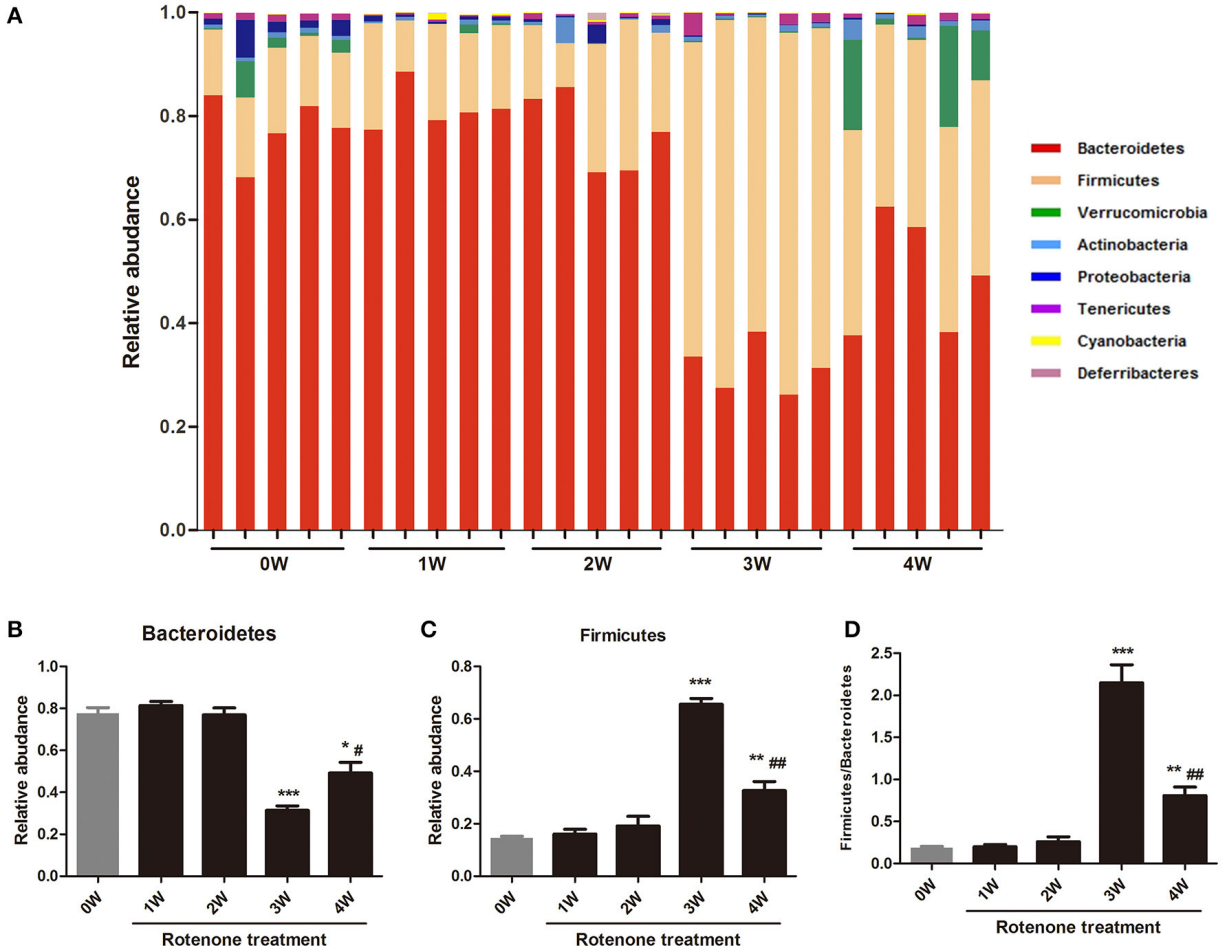
Relative abundances of fecal bacterial phyla among different times. **(A)** Each bar represents the relative abundance of OTU at the phylum level in the gut microbiota within a sample. **(B)** Relative abundance of Bacteroidetes among different times. **(C)** Relative abundance of Fibrobacteres among different times. **(D)** The ratio of Firmicutes/Bacteroidetes among different times. ^*^p < 0.05, ^**^p < 0.01, ^***^p < 0.001 vs. 0W. ^#^*p* < 0.05, ^##^*p* < 0.01 vs. 3W.

The gut bacterial community composition at the phyla level was shown in Figure 6A. Bacteroidetes, Firmicutes, Verrucomicrobia, and Proteobacteria represented more than 98% gene sequences. A distinct shift in the community profile was apparent among time points (Figure 6A) and based on PERMANOVA analysis, time was a significant source of variation (unadjusted *R*^2^ = 0.44195, *p* < 0.001). There was no significant difference of Verrucomicrobia, Proteobacteria and other phyla among different times. The Bacteroidetes was the most abundant, followed by Firmicutes at first 2 weeks, however after rotenone treatment, the relative abundance of Bacteroidetes was significantly decreased (*p* < 0.001) and of Firmicutes was significantly increased (*p* < 0.001) at 3 weeks in the rotenone-treated mice compared with the normal initial status (0 week). The relative abundance of Bacteroidetes (*p* < 0.05) and Firmicutes (*p* < 0.01) at 4 weeks were also different with 0 week (Figures 6B,C). The ratio of Firmicutes/Bacteroidetes was increased at 3 and 4 weeks of rotenone treatment (Figure 6D). The relative abundance of Bacteroidetes was increased (*p* < 0.05) and of Firmicutes was decreased (*p* < 0.01) at 4 weeks compared with 3 weeks. There was also a difference between 3 weeks and 4 weeks in the ratio of Firmicutes/Bacteroidetes (*p* < 0.01). Bacterial community at genus level with an abundant > 1%0, showing significant (*p* < 0.05) differences were shown in Table 1.

**Table 1:** Relative abundance of bacterial at genus level showing significant differences compared with 0W.

**Genus**	**0 W**	**1 W (*p*-value)**	**2 W (*p*-value)**	**3 W (*p*-value)**	**4 W (*p*-value)**
P__Firmicutes_Clostridium	0.0205	0.0001 (0.017^*^)	0.0002 (0.017^*^)	0.0009 (0.018^*^)	0.0011 (0.020^*^)
P__Proteobacteria_Sutterella	0.0053	0.0014 (0.027^*^)	0.0010 (0.020^*^)	0.0006 (0.008^**^)	0.0009 (0.014^*^)
P__Firmicutes_Lactococcus	0.0095	0.0001 (0.006^**^)	0.0001 (0.004^**^)	0.0004 (0.034^*^)	0.0009 (0.799)
P__Proteobacteria_Desulfovibrio	0.0060	0.0037 (0.198)	0.0011 (0.017^*^)	0.0002 (0.011^*^)	0.0003 (0.011^*^)
P__Firmicutes_Lactobacillus	0.0274	0.0476 (0.202)	0.0700 (0.205)	0.5834 (0.000^***^)	0.1493 (0.014^*^)
P__Bacteroidetes_Paraprevotella	0.0033	0.0006 (0.000^***^)	0.0031 (0.931)	0.0020 (0.016^*^)	0.0205 (0.107)
P__Actinobacteria_Adlercreutzia	0.0051	0.0022 (0.097)	0.0011 (0.004^**^)	0.0048 (0.825)	0.0082 (0.278)

*Highlighted*:

* *p < 0.05*,

** *p < 0.01*,

*** *p < 0.001 vs. 0 W*.

### Correlation of fecal microbiota with gastrointestinal and motor functions

Spearman correlations (Table 2) were applied to associate the differentially abundant genus of rotenone-exposed and control (0 week) groups. The genus of Desulfovibrio was positively associated with stool weight, stool water content, locomotor activity, and inversely correlated with climbing time. The genus of Lactobacillus was inversely correlated with stool weight, stool water content, locomotor activity, and positively associated with climbing time. The genus of Clostridium and Adlercreutzia were inversely associated with stool weight. The genus of Sutterella has no correlation with stool weight but positively associated with stool water content and motor functions.

**Table 2:** Correlation matrix (Spearman) of differentially abundant genus with stool weight, stool water content, locomotor activity, and climbing time.

**Variables**		**Clostridium**	**Sutterella**	**Lactococcus**	**Desulfovibrio**	**Lactobacillus**	**Paraprevotella**	**Adlercreutzia**	**Stool weight**	**Water content**	**Locomotor activity**	**Climbing time**
Stool weight	*r*	−0.419^*^	0.375	−0.392	0.646^**^	−0.608^**^	−0.312	−0.446^*^	1.000	0.718^**^	0.338	−0.498^*^
	Sig.	0.037	0.064	0.052	0.000	0.001	0.130	0.025	–	0.000	0.099	0.011
Water content	*r*	−0.156	0.422^*^	−0.053	0.662^**^	−0.658^**^	−0.149	−0.305	0.718^**^	1.000	0.381	−0.423^*^
	Sig.	0.456	0.036	0.801	0.000	0.000	0.476	0.138	0.000	–	0.060	0.035
Locomotor activity	*r*	−0.042	0.496^*^	−0.056	0.408^*^	−0.522^**^	−0.076	−0.165	0.338	0.381	1.000	−0.636^**^
	Sig.	0.842	0.012	0.790	0.043	0.007	0.719	0.429	0.099	0.060	–	0.001
Climbing time	*r*	−0.045	−0.507^**^	0.262	−0.496^*^	0.622^**^	0.278	0.202	−0.498^*^	−0.423^*^	−0.636^**^	1.000
	Sig.	0.829	0.010	0.206	0.012	0.001	0.179	0.334	0.011	0.035	0.001	–

*Highlighted*:

* *p < 0.05*,

** *p < 0.01*.

### Predictive functional (PICRUst) analysis

**Figure 7:**
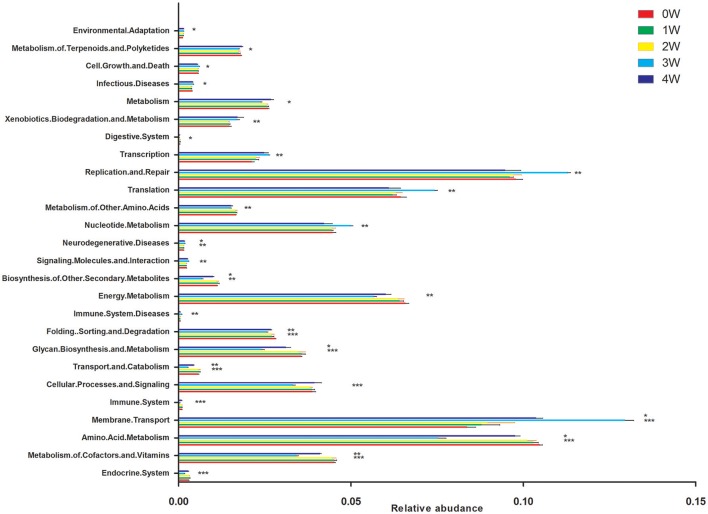
Predictive Functional (PICRUSt) analysis at level 2. ^*^p < 0.05, ^**^p < 0.01, ^***^p < 0.01 vs. 0W.

Predictive assessment of the microbial community functional potential (PICRUst) was used to predict the abundance of Kyoto Encyclopedia of Genes and Genomes (KEGG) pathways based on the 16S rRNA gene sequencing data. Forty-one KEGG pathways from the level 2 analysis were identified. There was no difference between 1 week or 2 weeks and initial status. Compared with initial status (0 week), there were 11 pathways predicted as enriched in the fecal microbiome of rotenone treated mice at 3 weeks, including Membrane Transport, Immune System Diseases, Signaling Molecules and Interaction, Neurodegenerative Diseases, Nucleotide Metabolism, Translation, Replication and Repair, Transcription, Xenobiotics Biodegradation and Metabolism, Infectious Diseases and Cell Growth and Death pathways. Fourteen pathways were lower including Endocrine System, Metabolism of Cofactors and Vitamins, Amino Acid Metabolism, Immune System, Cellular Processes and Signaling, Transport and Catabolism, Glycan Biosynthesis and Metabolism, Folding Sorting and Degradation, Energy Metabolism, Biosynthesis of Other Secondary Metabolites, Metabolism of Other Amino Acids, Digestive System, Metabolism, Metabolism of Terpenoids, and Polyketides pathways. Environmental Adaptation, Neurodegenerative Diseases and Membrane Transport pathways were enriched, Biosynthesis of Other Secondary Metabolites, Folding Sorting and Degradation, Glycan Biosynthesis and Metabolism, Transport and Catabolism, Amino Acid Metabolism and Metabolism of Cofactors and Vitamins pathways were lower at 4 weeks compare with initial status (Figure 7).

## Discussion

Parkinson’s disease is a progressive neurodegenerative disorder whose etiology is not understood. Recently studies have demonstrated gut microbiota is related to PD and clinical phenotype (Sampson et al., 2016; Scheperjans, 2016). Here we present a longitudinal study to evaluated the effects of oral rotenone treatment on the change of fecal microbiota using deep sequencing and its relevance to the pathologic processes of PD. Interestingly, we observed dynamic alterations in fecal microbiota that were correlated with the changes in the PD pathologic processes induced by rotenone. What is important, the alteration of fecal microbiota preceded the major motor dysfunction and CNS pathology of PD.

Rotenone is a mitochondrial complex I inhibitor that has been largely used to model PD in animals. Intragastric administration of rotenone during 4–6 weeks could reproduce the progression of PD pathology in mice. One recent report suggested that oral rotenone administered at 5mg/kg for 1.5 months induced α-synuclein aggregates in the ENS. Chronic oral treatment by rotenone for 4 weeks induced decrease in stool frequency and replicates neurodegeneration of the substantia nigra. Another study reported chronic infusion of rotenone for 22–28 days induced ENS dysfunction and delayed gastric emptying (Pan-Montojo et al., 2010; Tasselli et al., 2013; Klingelhoefer and Reichmann, 2015). Our results show that environment-contact administration of rotenone can reproduce the main behavioral and neuropathological features of human Parkinsonism. These alterations are sequential, treatment time dependent and accompanied by inflammatory signs in the colon and motor dysfunctions, which makes the model well-suited for the assessment of pathogenic pathways. Epidemiological evidence has linked environmental chemicals exposure to the incidence of PD (Brown et al., 2006; Hatcher et al., 2008). A few recent studies have documented the effects of environmental chemicals on gut microbiome community structures (Choi et al., 2013; Narrowe et al., 2015). However, the functional impacts of environmental chemicals on gut microbiome are not well-established. It has been reported Diazinon exposure perturbed the gut microbiome community structure, functional metagenome, and associated metabolic profiles (Gao et al., 2017). So far, there is a paucity of knowledge regarding the effect of rotenone on fecal micobitoa. Given the important roles of the gut microbiota in regulating normal physiological functions, environmental toxicant-induced functional perturbations of the gut microbiota may lead to or exacerbate their toxicity. In our study, we characterized the association between fecal microbiota and PD by using environment-contact administration of rotenone which mimicked the common ways of rotenone entering the human body from a longitudinal study. We summarized the key biochemical, histological, behavioral features and fecal microbiota in the process of PD.

As the Table 3 shown, rotenone-exposed animals started showing constipation at 3 weeks prior to motor deficits (4 weeks), indicating GI dysfunction preceding motor deficits. We also observed enhanced α-synuclein protein expression in the colon of rotenone-exposed mice before in the brain, which consistent with Braak’s hypothesis. Importantly, microbiota dysbiosis (from 3 weeks) in the feces was preceded the neuropathological hallmarks of PD (loss of dopaminergic neurons in the substantia nigra and α-synclein accumulation).

**Table 3:** Summary of key behavioral features, biochemical, histological and fecal microbiota change with time.

**Times**	**0 week**	**1 week**	**2 weeks**	**3 weeks**	**4 weeks**
GI dysfunction	No	No	No	Yes	Yes
Motor dysfunction	No	No	No	No	Yes
Gut inflammation	No	No	No	Yes	Yes
α-syn in colon	No	No	No	Yes	Yes
α-syn in brain	No	No	No	No	Yes
TH reduce in SN	No	No	No	No	Yes
Fecal microbiota dysbiosis	No	No	No	Yes	Yes

Keshavarzian et al. and Scheperjans et al. studies both found a significant difference in β-diversity between PD and control fecal samples which consistent with our PD animal model results (Keshavarzian et al., 2015; Scheperjans et al., 2015). Keshavarzian et al. study found an increased in α-diversity in PD patients than control in fecal samples but not in mucosa samples. No statistically significant differences were found with respect to α-diversity in Scheperjans et al. study. While, our 16S rRNA gene sequencing results found lower α-diversity in the model group (3 weeks) than the normal initial status (0 week) which can be seen in other animal model of diseases, including colorectal cancer and hypertension (Zhu et al., 2014; Yang et al., 2015). The Bacteroidetes and Firmicutes, consisting of more than 90% of all phylogenetic types in the human and mouse (Eckburg et al., 2005; Ley et al., 2006). Our data shows an aberrant microbial composition in the fecal of rotenone-exposed mice, with shifts observed in the main bacterial phyla. Phylum level analysis showed a clear alteration of the bacterial fecal community at 3 and 4 weeks compared with 0 week, characterized by a higher Firmicutes/Bacteroidetes ratio. The abundance of Firmicutes was elevated whereas the abundance of Bacteroidetes was reduced. Bacteroidetes was significantly reduced in fecal samples of PD patients (Unger et al., 2016). Several inflammatory conditions have been related to an increase in the Firmicutes/Bacteroidetes ratio such as inflammatory bowel diseases (IBDs) (Frank et al., 2007), obesity (Turnbaugh et al., 2008), and diabetes mellitus type 2 (Remely et al., 2014). Gut dysbiosis in hypertension is also characterized by an increased Firmicutes/Bacteroidetes ratio (Robles-Vera et al., 2017). Inconsistently with our results, a trend toward a lower Firmicutes/Bacteroidetes ratio in PD patients was observed between PD and control groups in Keshavarzian et al. study (Keshavarzian et al., 2015). For one thing, the variability between our studies can be introduced through different sequencing techniques, Keshavarzian et al. study sequenced the V4 region, while we sequenced the V4 and V5 regions, for another, Keshavarzian et al. study is based on confirmed PD patients, while we did a serial analysis of the fecal microbiota in animal model, the mice at 3 weeks and 4 weeks may represent the pre-motor or early stage of PD. Actually, in our study a lower Firmicutes/Bacteroidetes ratio was found between 4 weeks and 3 weeks.

Furthermore, we discovered that the relative abundance of the genus Clostridium and Sutterella were decreased in rotenone-treated groups along the time, while Lactococcus was decreased but return to normal after 4 weeks of rotenone exposure. The genus of Desulfovibrio was decreased from 2 weeks, Lactobacillus was increased from 3 weeks of rotenone treatment compared with the initial status. When looking at the correlations, the genus of Lactobacillus was inversely correlated with gastrointestinal and motor functions, the genus of Desulfovibrio was positively correlated with gastrointestinal and motor functions. Sutterella has no correlation with stool weight but was positively associated with stool water content and motor functions. Clostridium and Adlercreutzia were inversely correlated with stool weight. Our results confirmed the reported elevated level of Lactobacillus (Hasegawa et al., 2015; Hill-Burns et al., 2017) and lower level of Clostridium (Hasegawa et al., 2015) in PD patients. The increased Lactobacillus was also observed in diabetes mellitus type 2 (Sato et al., 2014) and the constipation-type irritable bowel syndrome (Malinen et al., 2005). Clostridium clusters is implicated in the maintenance of mucosal homeostasis, promoting Treg cell accumulation and prevention of IBD (Sokol et al., 2009; Atarashi et al., 2011). The lower level of Desulfovibrio is associated with increased gut permeability (Zhang-Sun et al., 2015). Sutterella is decreased in autism and new-onset Crohn’s disease patients (Williams et al., 2012; Gevers et al., 2014).

Nevertheless, it remains unclear as to how microbiota dysbiosis could be related to the pathologic processes of PD. Using mice that overexpress α-synuclein, Sampson et al. reported that gut microbiota are required for motor deficits, microglia activation, and α-synuclein pathology (Sampson et al., 2016). Colonic bacteria are a primary source of proinflammatory bacterial products, and gut microbiota dysbiosis may initate immune activation at the very least amplify and exacerbate an ongoing inflammatory process (Ivanov and Honda, 2012). In our results we observed higher expression of inflammatory cytokine and TLR2 in the colon at 3 weeks and 4 weeks in rotenone-exposed animals. From our results we could not get the conclusion that the increased expression of α-synuclein in the gut is the consequence of altered fecal microbiota. However, excessive stimulation of the innate immune system resulting from gut microbiota dysbiosis may accelerate extensive α-synuclein aggregation in the gut. The α-synuclein aggregation and TH degeneration in the brain were not accompany with enhanced inflammatory response in the brain, it is believed that marked glial activation may not be accompanied by dopaminergic neuron degeneration in chronic rotenone administration model (Inden et al., 2007), other pathways such as neuroendocrine and direct neural mechanisms may play a role in the fecal microbiota dysbiosis-mediated α-synuclein aggregation and TH cell death in the brain. From our results, we proposed that fecal microbiota dysbiosis may contribute to rotenone toxicity or at least accelerate the process of PD.

There are still some limitations in our study. One of the limitations is that bacterial changes were only measured in fecal samples. We also need to provide more detailed information concerning mucosa-associated microbiota. Another possible limitation is the statistic power of five samples per group may not be high for 16S rRNA sequencing and a larger number of samples should be utilized in further studies. Thirdly, this study was a longitudinal study, we cannot completely exclude that time may have affected our results, however, in studies of adult laboratory mice in which diet and general environmental conditions are controlled, the murine fecal microbiome shows a relative high degree of stability (Zhang et al., 2010; Schloss et al., 2012).

Overall, our results bring a new insight in the role of microbiota in PD. Gut microbiome perturbation may contribute to rotenone toxicity in the initiation of PD. Novel therapeutic options aimed at modifying the gut microbiota composition in PD patients may influence the initial step of the following cascade of neurodegeneration in PD.

## Author contributions

XY and YQ: performed molecular biology studies; XY: wrote the manuscript. SX: was involved in the data analysis; YS was responsible for statistical analyses; QX: designed the study, provided financial support and revised the manuscript.

### Conflict of interest statement

The authors declare that the research was conducted in the absence of any commercial or financial relationships that could be construed as a potential conflict of interest.
